# How Knowledge-Hiding Behavior Among Manufacturing Professionals Influences Functional Interdependence and Turnover Intention

**DOI:** 10.3389/fpsyg.2021.723938

**Published:** 2021-09-27

**Authors:** Lalatendu Kesari Jena, Deepika Swain

**Affiliations:** School of Human Resource Management, XIM University, Bhubaneswar, India

**Keywords:** knowledge hiding, human capital, interpersonal equation, collaboration, behavioral vulnerabilities

## Abstract

**Objectives:** Knowledge hiding is inappropriate behavior of employees at the workplace that makes the entire organization suffer a subtle yet significant loss. Lack of sharing makes the journey of learning an arduous process. This, in turn, gives rise to a series of uncivil behaviors, hence resulting in a decrease of functional interdependence (FI). The cascading result toll is a turnover intention (TI), resting only after turnover—an actual separation from the employer. Statistical analysis of the empirical data collected depicts the intensity of influence of FI and TI as a result of the knowledge-hiding behavior.

**Methods:** Three hundred sixty-three executives employed in three public and two private manufacturing organizations in eastern India were the respondents in our study. To analyze the difference in variables of the study, a *t*-test was carried out. The statistical findings suggest no significant difference among study variables. This specifies that, despite a considerable difference in levels of management, there was no significant difference in perceiving workplace incivility, knowledge-hiding behavior, FI, and TI items of our instruments.

**Results:** Correlation findings show a negative association between workplace incivility and functional interdependence (*r* = −0.37 when the value of *p* is <0.01) and a positive association among workplace incivility and turnover intention (*r* = 0.32 when the value of *p* is <0.01). The condensed effect of workplace incivility (β = −0.59 when the value of *p* is <0.001) along with an important presence of knowledge-hiding behavior (β = −0.68 when the value of *p* is <0.01) when the dependent variable is FI indicates that knowledge-hiding behavior is mediating a partial association among workplace incivility and FI. Similarly, the effect of workplace incivility (β = 0.43 when the value of *p* is <0.01) is decreased when the impact of knowledge-hiding behavior (β = 0.66 when the value of *p* is <0.001) was sizeable with TI being the dependent variable.

**Conclusion:** The effect of knowledge hiding is inversely proportional to FI, whereas sharing has a direct relation with TI. An exhaustive data sample and a rigorous statistical analysis may give a clear picture of the amount of impact of TI and FI due to the lack of knowledge sharing and/or knowledge hiding.

## Introduction

Manufacturing plays a pivotal role in the economic development of developing countries. The three-sector model (Fisher, 1945) of economy classifies manufacturing as a secondary sector comprising the series of industry-related group of tasks from the customer to the factory in a cyclical way (Garetti and Taisch, 2012), making it an indispensable part of the economy. With the passage of time, the manufacturing sector updates itself with the technological advancements, hence achieving increased efficiency, reduced cost, and mass production (Agwu and Bessant, 2021). The undeniable contribution of the sector is making an impact on the future of our society and the planet at large. Firms aim at achieving better economic sustainability, coupled with profit-oriented progression, return on investments, and business stability for a longer duration). The wealth-maximization goal makes the manufacturing industry the epicenter of natural and human capital (Haraguchi et al., 2017). Manufacturing industries have helped in eradication of unemployment and poverty. This hints at the labor-intensive nature of the industry. Human capital is the most invaluable resource although it may be exposed to many behavioral vulnerabilities, such as interpersonal equations at the workplace, different hygiene and motivation factors, and the synergistic balance of interpersonal-functional dependence (Kim, 2021).

In today’s “knowledge = power” world, experience, understanding, previous exposure, and important lessons learned over a period of time are considered no less than intellectual property (Butt, 2021). Patenting it so as to prevent its usage by another without giving due credit to the original owner is the silent salient practice at job places. The “I suffered and learned; let the other person suffer too” approach is ideally to be replaced with “knowledge shared = power multiplied” (Su, 2021). Cooperation, collaboration, empathy, mentoring, sharing—such words are mere parts of a company’s values webpage. It looks more like bait to attract the best and freshest talents to the world of the workplace. To fresh hires, these glorified words and their underlying real intentions act as a fatal potion, poisoning the tender mind with huge repercussions. The intention of this research is to throw light on the area of concern, the knowledge-hiding behavior. Establishment of a symbiotic relationship is possible only when both the parties of any communication or contract know the benefits and consequences. Thorough study of work behavior, the just-in-time supply of the right input at the right place, will ensure the right behavior. This ensures the flow of human capital to reach the pinnacle of profit: financial, physical, and psychological.

Understanding of an unthoughtful act of knowledge-hiding behavior will answer to the tip-toeing consequences of one of its brainchildren: workplace incivility. The functional interdependence (FI) makes the organization stable and composed in the face of any crisis. Victory over a crisis makes the employee acknowledge the strength of togetherness, hence nullifying the concept of parting with the company, i.e., low turnover intention (TI) (Siachou et al., 2021).

## Review of Literature

### Workplace Incivility and FI

Interdependence is the heart of every organization. Organizations have representatives—individual workers, small groups of workers, or business units—that cater to their unique contribution as a significant part of the overall work and, hence, are closely linked with each another by the master thread of interdependence (Su, 2021). Interdependence is a central concept to determine the agility of an organization. Functionally connected units with their aligned goals need to work toward the undisputed goal of the company (Gagne et al., 2019a,b). The modern era of technology, which can track every single lub-dub of the heart, measures each contribution of an employee in the minutest detail possible so as to ensure proper compensation in terms of short- and long-term incentives (Yoon et al., 2021). Incentivization has raised not only the bars of efficiency and competition, but also heightened the importance of knowledge sharing. FI is a necessity, and resultant knowledge hiding is a truth. The very existence of lack of FI is threatening to an organizational unit (Rezwan and Takahashi, 2021). We experience it in many places in which knowledge-hiding behavior or practice throttles many invaluable practices or art. Many cultural art forms, unique artisan techniques, invaluable crafts eloped into the golden future as a result of sheer knowledge hiding.

The revolutionary war song’s line: “United we stand, divided we fall,” states the current scenario in which individuals are in a state of war among each other—it is a war of knowledge. The more knowledge, the more incentives and more power. The reluctance to share experience forces individuals to indulge in incivility (Welbourne and Sariol, 2016) so that a needy employee cannot think of approaching them in the first place to ask for any mentorship, guidance, or information. Small yet subtle workplace mistreatment is faced by around 96% of employees (Porath and Pearson, 2010). This troubling phenomenon is identified by many researchers like us and is in the path of figuring out the establishment of FI and uprooting workplace incivility postunderstanding of the existence of such behavior (Strik et al., 2021). Workplace incivility is an uncivil, low-intensity, deviant behavior having an insignificant ambiguous intent to harm someone. As compared with many heinous and cruel deeds at the workplace, incivility is considered light, but it is present at every organization (Porath and Pearson, 2013). These sets of negative behaviors come from a varied number of masquerades; the loudest is a bully and least is a sycophant.

Workplace incivility causes tremendous amounts of mental stress among employees (Cortina and Magley, 2009) despite it being the most insignificant form of workplace deviance (Porath and Pearson, 2012). Cascading incivility is the root cause of severe job attitude-related concerns (Lim and Cortina, 2005), deteriorating physiological health (Lim et al., 2008), and challenges to mental health (Miner et al., 2012). The worst sufferers of incivility report higher burnout with respect to their jobs (Miner-Rubino and Reed, 2010). Moreover, the damaging side effects of workplace incivility barge into the personal space of employees, leading to a highly magnified work–family imbalance (Lim and Lee, 2011) and highly compromised satisfaction in married life (Ferguson, 2012). The unwelcoming ramifications of work incivility are the unuttered prime reasons for diluted and detrimental FI. The research findings, hence, validate the literature review making us propose the following:

H_1_: Workplace incivility is negatively related to functional interdependence.

### Workplace Incivility and TI

Appropriate work ambience is blissful; on the other hand, inappropriate workplace behavior not only negatively influences employees' intention to leave the organization, but sometimes may spoil the brand image of the company, creating long-lasting detrimental relapses (Chahar and Hatwal, 2021). Interpersonal mistreatment is the creator of a feeling of victimization, which leads to frustration (O'Reilly and Aquino, 2011) and acts as the seed of voluntary TI. The physical separation of an employee is called “turnover,” and it is merely the result of a sizable, long process. The intention of turnover is a step-by-step process of thinking, desiring, and planning to leave the current job (Lambert et al., 2010). The opportunistic behavior of keeping the current organization as a stop-gap arrangement is voluntary and increasingly, is a result of workplace incivility (Viotti et al., 2021). The ultimate destination of every turnover intention is turnover itself (Lambert et al., 2010). Workplace discriminant behavior sows the seed of an intention to leave the job; with the passage of time, if the incivility improves to civility, TI fades away, hence aborting the process. The continuation of incivility evolves to an active job search while being in the current organization (Paille' and Dufour, 2013). The deceitful act is the brainchild of workplace incivility. TI incurs a direct cost to the company in the form of human capital loss and an indirect cost in terms of potential loss of expert workforce, social linkages, and prospective clientele; decreased synergy among the inmates; exploitation of the existing workforce; and compromised employee morale, thereby entering into the vicious cycle of further turnover. Loss of rare intellectual capital is another potential result of TI.

In our study, we intend to prove our hypothesis of a positive correlation between workplace incivility and TI. We have hypothesized workplace incivility as an independent variable in our empirical investigation. The research journey enlightened our finding that TI is aggravated by workplace incivility. The cascading job dissatisfaction, psychological distress, and physical health deterioration are the damaging impacts of interpersonal maltreatment. Sooner or later, these impacts proliferate to result in TI, weighing the pros and cons of a new employer while waiting for the right time to leave the present company to demand the best at the new workplace, and the final result of bidding goodbye, i.e., the actual day of turnover. Workplace incivility–induced TI creates a flowchart of negative behaviors that may prove sizably dear to the company. Based on a review of the literature, we propose the following hypothesis:

H_2_: Workplace incivility is positively related to TI.

## Beyond the Direct Effect of Workplace Incivility

Fetching a livelihood is seldom the prime purpose of working for any individual (Mustika et al., 2019). It is the passion, interest, and strengths that drive the choice of zeroing down a workplace. Workplace civility and FI coupled with a knowledge-sharing environment (Wu et al., 2020) make an employee take pride in one's fate of being able to make the right choice of workplace. Contrastingly, workplace incivility, missing/compromising FI, and knowledge hiding make the workplace a compulsion to fetch a mere livelihood (Oliveria et al., 2021). The welcoming mentorship is the most important gift an employer can give to its employee. It many times starts weaving the invisible threads of belongingness in the mind of a new entrant. An improper welcome often is the result of a lack of leadership qualities of the senior person at the workplace. Employees have slipped into the clutch of patenting each lesson, making the sharing of knowledge, hence gaining, an impossible task. They want others to get the similar learning after the amount of toil they have undergone. When the learning is equally tough and challenging, gratitude goes missing at the workplace (Connelly et al., 2019). Tech giant Google duly understands the cost of work incivility in terms of turnover. The company has taken an ensuring step to formalize its approach to civil interaction among employees. It has categorized a list of prohibited behaviors as an attempt to control workplace incivility. The top provisions are “doxing” (revealing someone else's personal information), “trolling” and name-calling, and using “blanket statements” about certain groups of people. It rather ensures to encourage workers to “understand more, not be right.”

Emotions do not spontaneously erupt, but are unnoticeably programmed by changes in one's environment of exposure. Emotions at the workplace typically emerge from our social interactions, and in turn, our social interactions reflect and transmit our emotions at the workplace (Geddes et al., 2020). Joining a new organization is the period of palpitation, that creates impactful emotions for a lifetime. Information, experience, and expertise ideally constitute knowledge and play a pivotal role in the journey of an employee's workplace (Connelly et al., 2019). If not shared at the right time with the right vigor, it is seen as knowledge hiding. A continuous practice of knowledge hiding results in knowledge hoarding. This leads to a plethora of counterproductive workplace behaviors, and workplace incivility is one of them, which, in turn, mediates a lack of FI. Thus, we postulate the following hypothesis:

H_3_: Knowledge-hiding behavior mediates the relationship between workplace incivility and FI.

### Knowledge-Hiding Behavior and TI

Knowledge is empowered as the center of the creation of competitive advantage for any organization (Wang and Noe, 2010). Hence, hiding knowledge becomes crucial as it acts as a hindrance in transmitting the competitive edge of the organization to its employees. Extensive research in the area of knowledge exchange and its impact on employee morale reveals that imparting and exchanging knowledge and information boosts team spirit and improves organizational performance. On the other hand, knowledge hiding mediates a lack of trust. The absence of trust makes the workplace an all-time Darwinian plot; employees have to struggle every time for their existence, and the fittest alone survive. The course at the workplace becomes bitter, hence mediating uncivil behaviors. Knowledge sharing and combining as the flag bearers of knowledge transfer (Wang and Noe, 2010) get thoroughly crippled with knowledge-hiding behaviors. The chain of hiding grows stronger and stronger, giving birth to workplace incivility.

**Figure 1:**
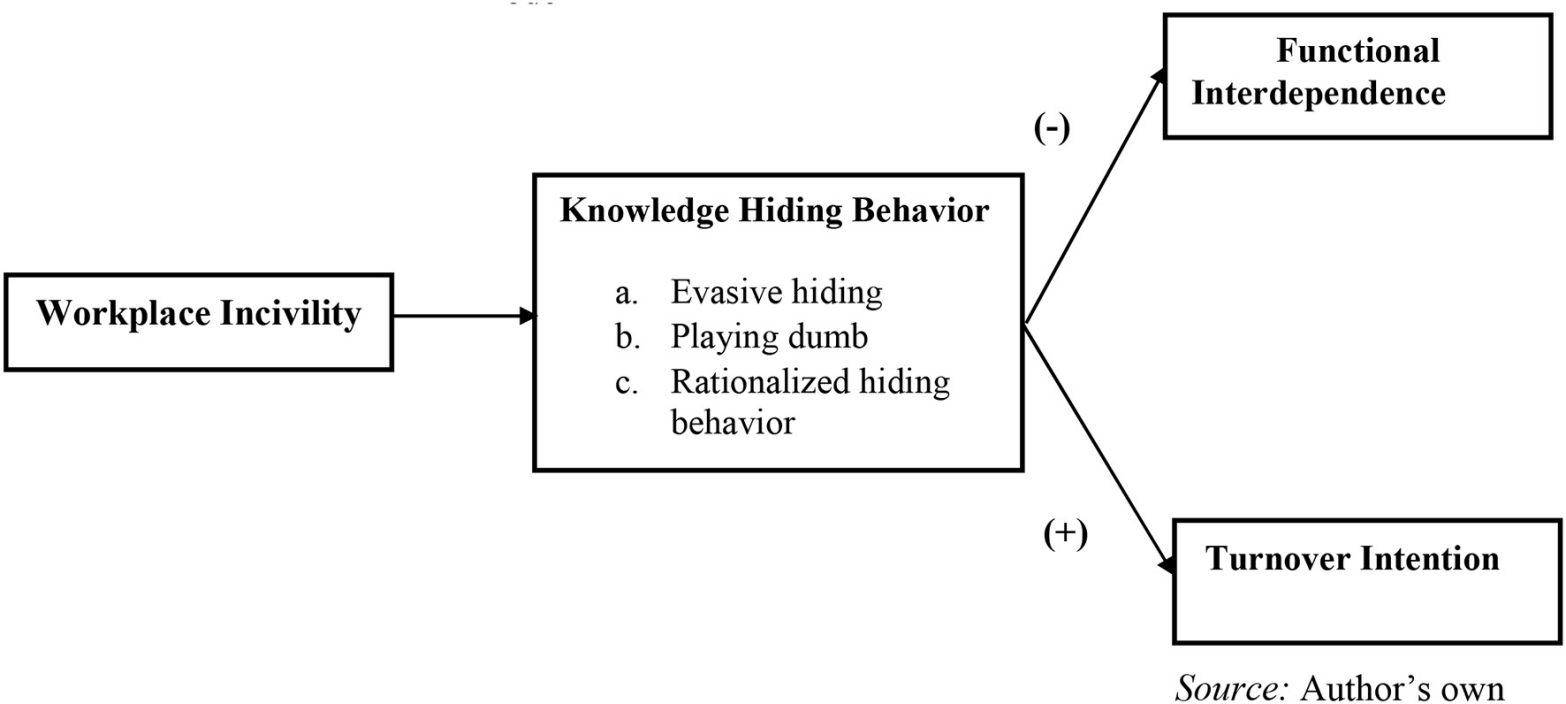
Hypothesized model.

In its various forms, workplace incivility dilutes job satisfaction (Viotti et al., 2021). It slowly builds a loss of comfort level with peers, fabricated conversation, workplace avoidance, and a sprouting desire for fetching a better workplace. Hence, it is highly essential to make employees' knowledge accessible to as many employees and departments as possible to construct a healthy organization (De Vries et al., 2006). Knowledge sharing may be better explained as the specific set of information and experience that may enable others to simulate the requisite problem-solving skills, generation of new idea pools along with the implementation of unique methods and processes (Wang and Noe, 2010). Extensive work done in this area unveils a positive association of both team and organizational productivity and performance with sharing and exchanging knowledge and information (Collins and Smith, 2006; Wang and Noe, 2010). Because work related to knowledge is valued significantly during the process of generating wealth in the recent global–local economy, internalizing the precursors of knowledge sharing has become an exponentially important concern (De Vries et al., 2006; Gagné, 2009; Frost et al., 2010). Therefore, we propose the following hypothesis, and the conceptual framework of the study is presented in Figure 1:

H_4_: Knowledge-hiding behavior mediates the relationship between workplace incivility and TI.

## Methods

### Samples and Procedures

Executives employed in three public and two private manufacturing organizations in eastern India were the respondents in our study. The chosen organizations are labor intensive, have a defined hierarchical structure of reporting, and are involved in producing high volumes of mechanical supplies to the consumer market. Formal approval from HR departments was obtained prior to conducting the questionnaire survey. An internal memo was made by the respective HRD/training departments to the executive respondents of the respective organizations with a request for voluntary participation in our pencil and paper mode of survey exercise. Confidentiality and anonymity were ensured to the executives for their participation. Questionnaires were coded with an identification number, and the initial set of questions were reserved to elicit demographic information about the sample respondents.

To begin, 570 executives, including shop floor engineers and managers, research and design engineers and managers, finance and payroll executives, managers from vigilance, marketing and purchase, and the quality department showed interest in participating in our survey. In the end, 437 filled-in questionnaires were sent to the HR departments of the respective organizations for onward collection by us. This provides a response rate of 76.6%.

Seventy-four filled-in questionnaires were rejected because of missing information, and thus, 363 (83%) valid responses were considered for further statistical analysis. The sample response comprises 114 (31.4%) female executives and 249 (68.6%) male executives with an average work experience of 6.3 years (SD = 2.84) in their present organization; 42.7% (*N* = 155) of sample executives were in private manufacturing units, and 37.4% (*N* = 136) of both private and public sector sample respondents belonged to the upper level of management. Of the total sample, 198 (54.54%) respondents got their education with a master's degree, and the rest had a university-level technical/non-technical degree education; 227 employees (subordinates) were reporting to 136 senior executives. The questionnaire was distributed in such a way that the subordinates did not know what questions were asked of the superiors and vice versa. This helped them to answer bias- and coercion-free.

We carried out a *t*-test to analyze the differences in variables between different positions of management, i.e., superiors and subordinates. The statistical findings suggest no significant difference among study variables across different levels. This specifies that despite the considerable differences in levels of management, there was not much of a significant difference in perceiving workplace incivility, knowledge-hiding behavior, FI, and TI items of our instruments.

## Measures

Incivility in the workplace was assessed by the “workplace incivility scale” developed by Cortina et al. (2001). It is an eight-item measure used to understand the sample respondents' experience of discourteous, impolite, or patronizing behavior from their immediate superior. The Cronbach alpha (α) of the scale is high, having a coefficient of 0.91 and, thus, demonstrating decent reliability and cohesiveness. A sample item from the scale is “Have you been in a situation where any of your superiors paid little attention to your statement or showed little interest in your opinion?” A Likert scale with five items ranging from “not at all” to “to a great extent” was used as a measurement tool.

Knowledge-hiding behaviors of executives adopted from was measured on a five-point Likert scale ranging from “not all” to “completely.” We adopted 12 items out of 21 capturing three dimensions of the scale (evasive hiding, playing dumb, rationalized hiding) to recognize the unwanted effect of possessing knowledge in a work atmosphere. The items of the instrument were appraised in a five-item Likert-type scale depicted as “not at all” to “to a great extent.” The reliability (α) for each subscale was 0.86, 0.88, and 0.81, and the overall coefficient of the scale was 0.86. Sample items include “I agree to help him/her but instead give him/her information different from what s/he wanted” (evasive hiding), “I pretend I do not know what s/he was talking about” (playing dumb), and “I explain that the information is confidential and only available to people on a particular project” (rationalized hiding).

The *TI* was measured with 30 items on the Multidimensional Turnover Intention Scale developed by Menezes et al. (2018). The scale comprises two dimensions (intrinsic and extrinsic) and captures the psychological forces that initiate sensible deliberation about one's organizational membership, that is, whether to continue or leave the organization. One of the items of the scale goes “I would move to another company if I were unhappy with different aspects of my current organization (team, leadership, working conditions, etc.), even aware that my salary would be lower at this other company.” Cronbach's alpha (α) of the instrument is high, having the coefficient of 0.93 and, thus, demonstrating decent reliability and cohesiveness. The items were by five-point Likert scale in a range of “strongly disagree” to “strongly agree.”

*FI* was assessed by a 6-point subscale designed by Alves and Lourenco (2017). The instrument captures the knowledge of roles, functions, tasks, and responsibilities of colleagues at work by gauging their ability to accomplish each other’s assignments at the time of need. The items of the instrument were appraised in a 5-item Likert-type scale depicted as “not at all” to “to a great extent.” A sample item of the scale is: “In my team, when someone is missing at work, the other group members have the knowledge to perform their tasks.” The coefficient (α) of the scale in the study is 0.94. The scale was measured for each subordinate by their immediate supervisor/reporting authority. The data generated through this instrument were matched with the self-reported workplace incivility, knowledge-hiding behavior, and TI measures. We obtained the support of a research assistant in this regard to match the self-reported data with their immediate reporting authority’s data. The data was cleaned by eliminating the identity information of the sample respondent and then was passed to the authors to carry out their statistical testing and analysis.

## Findings

Mean, S.D., and correlation among the study variables are presented in Table 1. The data on the constructs, such as workplace incivility, knowledge-hiding behavior and TI collected from employee surveys advocated adequate fit through confirmatory factor analysis. The variables were examined through structural equation modeling in AMOS 21.0. The correlation findings were used to examine both H_1_ and H_2_ of the study. The hypotheses that predicted workplace incivility and FI (negative association) and workplace incivility and TI (positive association) were both supported in their desired directions. Correlation findings show a negative association between workplace incivility and FI (*r* = −0.37 when the value of *p* is <0.01) and a positive association among workplace incivility and TI (*r* = 0.32 when value of *p* is <0.01).

**Table 1:** Statistics of Uni-variation and correlations between variables.

**Variables**	**Mean**	**S.D**.	**a**	**b**	**c**	**d**
a. Workplace incivility	3.62	0.54	***(0.91)***			
b. Knowledge-hiding behavior	3.71	0.63	0.44^**^	***(0.86)***		
c. Functional interdependence	3.24	0.49	−0.37^**^	0.56^**^	***(0.94)***	
d. Turnover intention	3.26	0.68	0.32 ^*^	−0.43^**^	−0.29^***^	***(0.93)***

*N = 363*;

* *p < 0.05*;

** *p < 0.01*;

*** *p < 0.001; diagonal (bold and italics) are the reliability values (α)*.

For examining the mediating effect of knowledge-hiding behavior on the association between workplace incivility and FI and between workplace incivility and TI, we adopted the three-step approach proposed by Baron and Kenny (1986). Initially, we regressed the mediator (knowledge-hiding behavior) on workplace incivility. In the next model of regression, the dependent variables (FI and TI) were regressed on the independent variable. Finally, with a statistically significant value on the second regression, the dependent variables were regressed on both the mediator as well as the independent variable. In addition, we also performed the Sobel test (Sobel, 1988) to understand the significance of the mediation made by knowledge-hiding behavior.

The findings are presented in Table 2 for both the dependent variables of our study (FI and TI). The condensed effect of workplace incivility (β = −0.59 when the value of *p* is <0.001) along with the importance of knowledge-hiding behavior (β = −0.68 when the value of *p* is <0.01), FI being the variable of dependence, indicated that partial mediation of the association between workplace incivility and FI is the bane of knowledge-hiding behavior partially. Sobel test (Sobel, 1988) findings reconfirmed the significant presence of the indirect effect (*z* = −2.12 when the value of *p* is <0.001). After including the knowledge-hiding behavior in the regression, findings clarified 6% additional variance in functional interdependence above the variance resulting solely due to workplace incivility. Similarly, the impact of workplace incivility (β = 0.43 when the value of p is <0.01) is decreased when the impact of knowledge-hiding behavior (β = 0.66 when the value of *p* is <0.001) was significantly visible when TI was the variable of dependence. The findings of the Sobel test (Sobel, 1988) also revalidate the significant importance of the indirect influence (*z* = 3.36 when the value of *p* is < 0.001) as knowledge-hiding behavior describes an 18% additional variance in the TI above the variance amounted by only workplace incivility. The said result findings support H_3_ and H_4_.

**Table 2:** Mediating role of knowledge-hiding behavior on the existing association between workplace incivility and functional interdependence, turnover intention.

**Variables (Dependent)**	**Findings of Sobel test**	**IV: Mediator**	**IV: DV**	**Mediator: DV**	**IV: DV (Controlled by mediator)**
Functional interdependence	−2.12^***^	0.47^***^	−0.62^**^	−0.68^**^	−0.59^***^
Turnover intention	3.36^***^	0.39^**^	0.84^**^	0.66^***^	0.43^**^

*Path values are β; N = 363; IV, Independent variable (workplace incivility); DV, Dependent variables (Functional interdependence, Turnover intention)*;

^*^p < 0.05;

** *p < 0.01*;

*** *p < 0.001*.

### The Mediating Role of Dimensions of Knowledge-Hiding Behavior

We carried out additional analyses to examine the independent mediating influence of the three dimensions that build the construct: knowledge-hiding behavior. Although write that knowledge-hiding behavior is a complement construct with 21 items that define “evasive hiding, lack of sharing, playing dumb, rationalized hiding, and knowledge hoarding,” others (e.g., Demirkasimoglu, 2016) consider the dominant influence of three dimensions—“evasive hiding, playing dumb, and rationalized hiding”—that make up the differential effect on knowledge-hiding behavior in an organizational setup. For a better understanding of how knowledge-hiding behavior influences the hypothesized association, we have once again conducted the mediation tests to independently assess the three dimensions suggested by Demirkasimoglu (2016).

The three-step process suggested by Baron and Kenny (1986) was used to examine the potential of each dimension of the behavior of hiding knowledge as mediators independently. In the first step, we tested the association between hiding evasively, hiding rationally, and playing dumb along with their impacts on workplace incivility (IV). Table 3 presents the regression findings. The results imply that all three dimensions, hiding evasively (β = 0.57 when the value of *p* is <0.01) and hiding rationally (β = 0.38 when the value of *p* is <0.01), were significantly related, and playing dumb (β = 0.44 when the value of *p* is <0.05) is positively related to workplace incivility. The second step of the process suggests that the dependent variable regresses on the fixed variable; this is stated earlier in Table 2.

**Table 3:** Mediating role of dimensions of knowledge-hiding behavior on the association between workplace incivility and functional interdependence, turnover intention.

**Mediating variables**	**Sobel test findings (Functional interdependence)**	**Sobel test findings (Turnover intention)**	**IV: Mediator**	**Mediator: Functional interdependence**	**IV: Functional interdependence (Controlled by mediator)**	**Mediator: Turnover intention**	**IV: Turnover intention (Controlled by mediator)**
Evasive hiding	−3.21^***^	3.43^***^	0.57^**^	−0.36^**^	−0.29^***^	0.42^**^	0.28^**^
Playing dumb	−2.26^**^	3.27^***^	0.44^**^	−0.11	−0.17^**^	0.39^***^	0.44^***^
Rationalized hiding	−3.47^**^	2.79^**^	0.38^**^	−0.19^***^	−0.46^***^	0.51^***^	0.22^***^

*Path values are β; N = 363; IV, Independent variable (workplace incivility); DV, Dependent variables (Functional interdependence, Turnover intention)*;

* *p < 0.05*;

** *p < 0.01*;

*** *p < 0.001*.

In the third and final step, the dependent variables (functional interdependence and turnover intention), were regressed on the dimensions (hiding evasively, hiding rationally, and playing dumb) of knowledge-hiding behavior with workplace incivility as well. The results show the significant effect of workplace incivility on FI (β = −0.29 when the value of *p* is <0.001, β = −0.17 when the value of *p* is <0.05, β = −0.46 when the value of *p* is <0.001) and TI (β = 0.28 when the value of *p* is <0.01, β = 0.44 when the value of *p* is <0.001, β = 0.22 when the value of *p* is <0.001) when evasive hiding, playing dumb, and rationalized hiding were introduced. Sobel test (Sobel, 1988) findings imply the indirect effect of all three dimensions of knowledge-hiding behavior on FI (*z* = −3.21 when the value of *p* is <0.001, *z* = −2.26 when the value of *p* is <0.05, *z* = −3.47 when the value of *p* is <0.01) and TI (*z* = 3.43 when the value of *p* is <0.001, *z* = 3.27 when the value of *p* is <0.001, *z* = 2.79 when the value of *p* is <0.01) were significant.

## Discussion and Implications

Statistical analysis of the research intention investigated how knowledge hiding leads to knowledge hoarding, which amounts to work incivility, in turn, amounting to low FI and high TI. An extensive study of more than 32 research articles, ranging from the past to the most recent, was done before formulating the hypothesis. A sample of 570 respondents included shop floor engineers and managers, research and design engineers and managers, finance and payroll executives, managers from vigilance, marketing and purchase, and the quality department showed interest participating in the survey, whereas 437 could actually make it. The response rate of 76.6% made it reach the next level of investigation. Gender bias error was given due importance with a 3:7 ratio of female to male respondents. The pedigree of the employee being yet another criterion of importance got its due place while choosing the sample respondents.

The framing of the hypotheses was simple and powerful to reach to a common man and a senior executive at the same tempo. The first hypothesis hovered around workplace incivility and FI. It proved that workplace incivility is negatively related to FI. The second hypothesis examined workplace incivility and TI, finally establishing the fact that workplace incivility is positively related to TI. Beyond the direct effect of workplace incivility, knowledge-hiding behavior was treated as mediating a conjuring relationship between workplace incivility and FI. Knowledge-hiding behavior and TI share a direct relationship. The study grew most interesting by the empirical evidence collected from the primary source. In the current scenario, we are learning to live with COVID post-COVID. The emotional aspect at the workplace has become increasingly crucial, especially in labor-intensive industries. The results of the study indicate a quick managerial redressal.

Empirical validation of the study supports the inverse relationship of workplace incivility to FI and direct relationship to TI. This depicts the clear intention of workplace humiliation. Low morale, suppressed self-esteem, and psychological depression lead to higher dependence on functional interdependence; sadly the interdependence offered becomes significantly low, adding to even more dependence and dissatisfaction. The team spirit dips, making the workplace the instigating place of frustration. Continuous low feeling leads to thinking of “flight” to escape from the situation as soon as possible, the birthplace of TI. A study of our sample respondent's responses unveiled that knowledge-hiding-triggered hoarding does not only delay the learning, but also initiates many irreversible changes at the subconscious level. Hence, we have statistically proved the significant role of knowledge-hiding behavior in mediating workplace incivility and TI with the help of hypothesis 4. The manager's concern may shift from hiding the knowledge to looking for the qualified recipient of this resource. This changed approach lets the benefits trickle down in the form of knowledge sharing, healthy TI, rewarding FI, and an escalated bottom-line of profits: long and short term, both.

## Limitations and Conclusion

Limitations are an inseparable part of our research findings, making it an active effort to address the current mediators and leaving a possibility for future updates in the findings by addressing the areas of concern. First, it being a behavioral study, the larger the sample size, the better the results may be. The initial 25% non-response from our sample size of 570 added to the limitation further. We feel, with a larger sample size, we could capture the result in a better way. Second, the hypothesis formation hovered around two dependent variables: FI and TI. A greater number of variables, such as lack of personal motivation, time pressure, promote turnover, and many more, may influence the result, giving yet another dimension to the research. Third, the mental state of the respondent while answering the questionnaire was different and, hence, may have made the answering process biased. A prefixed suitable timing for the entire process may make for better evaluation of the items in the questionnaire, hence limiting the rejected response to even less (for this research, the rejected questionnaire was 17%, i.e., 74). Fourth, the sector under consideration here was manufacturing, a labor-intensive wing of the economy. Knowledge sharing here saves time and energy; on the contrary, knowledge hiding multiplies the learning time. Other sectors may not share similar characteristic features. This might make our findings of little importance to other important sectors.

Despite these felt and stated limitations, the result of our study is attempting to make a significant contribution to workplace behavior in a number of ways. First, we provide a holistic understanding of the unexpressed behaviors of importance at the workplace. Post-pandemic, companies have entered into the privacy of the employees by tracking them every second with the help of different software, such as Prodoscore, TransparentBusiness, and many more. Prevalence of knowledge hiding can worsen the situation as the relationship between the employee and employer is already at the verge of reaching the elasticity limit. Second, we explore the conjuring effects of workplace incivility by the identification of verbal or non-verbal clues. This makes the workplace hostile, intimidating, and isolating. Riding on the comfort zone of fear of the employees, playing with their hopelessness and helplessness can ruin an entire empire hassle-free. Hence, understanding the severity of workplace incivility as a result of knowledge hiding is of paramount importance to managers. It may give the managers an immediate future direction to motivate their first customers, *their employees*, to devise their own tool kit to detach and disengage themselves from any uncivil behavior by stopping becoming a doormat or punching bag at the workplace. Finally, a closer examination of the hypothesis about the interrelation and impact of workplace incivility, taskforce interdependence, and TI share their nexus to the knowledge-hiding behavior at the workplace. Expansion of the comfort zone may call for dynamic changes in the culture of organizations. At a time when we all are fighting against a physical pandemic, the coronavirus, all organizations need to first fight against the silent pandemic of knowledge-hiding behavior. Hiding of knowledge, a result of the power gradient, clearly demonstrates in unsaid words the lack of respect. This may prove fatal to organizations resulting in reduced productivity and increased turnover, impacting the bottom line negatively due to unhappy and unproductive employees. Our research is a small effort to make our workplace a little more amicable and adorable by breaking our silence, reversing the epidemic of knowledge hiding to knowledge sharing.

## Data Availability Statement

The raw data supporting the conclusions of this article will be made available by the authors, without undue reservation.

## Ethics Statement

Ethical review and approval was not required for the study on human participants in accordance with the local legislation and institutional requirements. The patients/participants provided their written informed consent to participate in this study.

## Author Contributions

LJ designed the model, performed the data collection and analysis, and framed the limitations of the study. DS has made an extensive literature review, collating the analysis findings, and edited the final manuscript. All authors contributed to the article and approved the submitted version.

## Conflict of Interest

The authors declare that the research was conducted in the absence of any commercial or financial relationships that could be construed as a potential conflict of interest.

## Publisher's Note

All claims expressed in this article are solely those of the authors and do not necessarily represent those of their affiliated organizations, or those of the publisher, the editors and the reviewers. Any product that may be evaluated in this article, or claim that may be made by its manufacturer, is not guaranteed or endorsed by the publisher.
